# The WCIRDC 2023: Concepts of insulin resistance

**DOI:** 10.1111/1753-0407.13552

**Published:** 2024-03-22

**Authors:** Zachary Bloomgarden

The 21st annual World Congress on Insulin Resistance, Diabetes and Cardiovascular Disease, held in Los Angeles, California on December 7–9, 2023, included 69 presentations spanning a myriad of aspects of diabetes and its complications, of atherosclerosis, of renal disease, of liver disease, and of novel therapeutic approaches. For this summary, we will focus on presentations illustrating the current understanding of insulin resistance.

Giving an overview of insulin resistance, Ralph DeFronzo reviewed the complex pathways involved in glucose‐handling. A total of 5%–10% of ingested glucose is ultimately removed in adipocytes, and the remainder in skeletal muscle. The insulin signal that regulates glucose disposal is associated with changes in adipocyte free fatty acid release, as well as in local vasodilatation via nitric oxide. In type 2 diabetes (T2D) all of these are abnormal.^1^ T2D is associated with a defect in muscle glycogen synthesis and glucose oxidation,^2^ with impaired muscle glycogen synthase, pyruvate dehydrogenase, and hexosekinase.^3^ The insulin signaling pathway involved in activating glucose transport is also involved in activating nitric oxide synthase, with both reduced in T2D, whereas several proinflammatory/atherosclerotic pathways, involving mitogen‐activated protein kinase (MAPK) and the nuclear receptor small heterodimer partner, show unrestricted insulin response in T2D leading to vascular smooth muscle growth and inflammation. Lean offspring of two T2D parents who have normal glucose tolerance have hyperinsulinemia and show levels of insulin resistance similar to those of their parents, with the same defect in insulin receptor substrate and the same overactivity the MAPK pathway.^4^ Hepatic glucose output is increased in T2D,^5^ with the dose–response curve of hepatic glucose production vs portal insulin levels shifted to the right and evidence of decreased adipocyte insulin response,^6^ and muscle capillary bed insulin‐induced vasodilatation^7^ also is impaired in T2D. DeFronzo observed that thiazolidinediones reverse all of these molecular defects in T2D, suggesting that use of these agents should be considered more strongly in clinical treatment.

Sam Klein asked which comes first, beta cell dysfunction, hyperinsulinemia, or insulin resistance? Answering this seemingly straightforward question, he observed, requires better understanding of the many underlying interrelationships. In a study comparing lean, normoglycemic persons with obese persons having varying degrees of glycemia, he found that the hyperinsulinemia of obesity is primarily associated with increased insulin secretion, as well as with reduction in cell surface insulin receptors in tissues responsible for insulin clearance.^8^ In this view, Klein considered that “it's the beta cell which tips you over into T2D,” citing studies showing that, with impaired glucose tolerance and diabetes, insulin secretion abnormality reflects impaired K‐ATP channel density.^9^ However, he also reviewed a study of 24‐hour insulin infusion causing physiologic hyperinsulinemia leading to impaired insulin‐stimulated glycogen synthase activity and muscle insulin sensitivity^10^ and a study showing that individuals with higher insulin secretion rates progress to glucose intolerance.^11^ Furthermore, a 6‐month study of treatment with the K‐ATP channel closing agent diazoxide in conjunction with weight loss improved hyperinsulinemia and insulin sensitivity.^12^ His studies of dietary^13^ and bariatric surgery‐induced^14^ weight loss suggest that the effect of weight loss on glucose‐stimulated insulin secretion “depends on where you start,” leading to his proposal that obesity stimulated increase in insulin secretion should be considered the initiating cause of insulin resistance, leading to progressive dysglycemia.

Finally, Paul Zimmet reviewed the “historical milestones” leading to our present understanding of insulin resistance. He started with the *Lancet* publication in 1936 of Harold Himsworth's study differentiating insulin sensitive vs resistant diabetes.^15^ In 1949, Rachmiel Levine proposed that “insulin acts upon the cell membranes of certain tissues in such a manner that the transfer of hexoses (and perhaps other substances) from the extracellular fluid into the cell is facilitated,”^16^ and, in the same year, Joseph Bornstein developed the first insulin bioassay, confirming with R. D. Lawrence the existence of two types of diabetes “with and without available plasma insulin.”^17^ In 1960, the paper by Yalow and Berson describing the insulin immunoassay was finally published,^18^ after battling recommendations beginning in 1955 made by reviewers and by the editor of the *Journal of Clinical Investigation* to reject it. A number of important studies by Peter Bennett and colleagues began with the recognition of highly insulin‐resistant diabetes among Pima Indians initially published in 1971,^19^ with Zimmet subsequently finding similar evidence of highly prevalent insulin resistant diabetes among Pacific islanders,^20^ leading to his coining the term “Coca Colonization” to described adverse effects of adoption of Western lifestyles. Zimmet described what he termed “Starling's curve of the pancreas” in 1978, observing, “Compensatory hyperinsulinism may for some time prevent severe hyperglycaemia. However, once a critical level of hyperglycaemia is reached (ie, 2 h plasma glucose of approximately 280mg/100ml) beta cell function begins to fail and there is more rapid progression of hyperglycaemia. Thus, individuals might for some time remain in the ‘compensated’ phase of moderate hyperglycaemia and then move rapidly to a ‘decompensated’ phase of severe hyperglycaemia with a ‘falling’ insulin response.”^21^ Gerald Reaven's 1988 Banting Lecture described the constellation of risk factors based on insulin resistance as being more important than cholesterol in the development of cardiovascular disease,^22^ and Zimmet emphasized the multiple contributions of Jesse Roth in the understanding of the role of the cell membrane insulin receptor beginning in 1991.
